# Death from Inhaling a Sponge

**Published:** 1901-04-15

**Authors:** 


Death from Inhaling a Sponge.—A young man in England was anesthetized by a dentist and eleven teeth extracted, an assistant left a sponge, used in absorbing the blood, in the mouth and by a deep inspiration the sponge was carried into the throat and stopped the air passage so that the patient died from asphyxia. The usual methods of resuscitation were employed, but without avail as the operator did not know what the cause was. It might be well for dental surgeons to adopt the principle of counting their sponges as the abdominal surgeons do. Better not to use them.

